# Find_tfSBP: find thermodynamics-feasible and smallest balanced pathways with high yield from large-scale metabolic networks

**DOI:** 10.1038/s41598-017-17552-2

**Published:** 2017-12-11

**Authors:** Zixiang Xu, Jibin Sun, Qiaqing Wu, Dunming Zhu

**Affiliations:** 1 National Engineering Laboratory for Industrial Enzymes and Tianjin Engineering Center for Biocatalytic Technology, Tianjin Institute of Industrial Biotechnology, Chinese Academy of Sciences, Tianjin, 300308 China; 2 Key laboratory of systems microbial biotechnology, Tianjin Institute of Industrial Biotechnology, Chinese Academy of Sciences, Tianjin, 300308 China

## Abstract

Biologically meaningful metabolic pathways are important references in the design of industrial bacterium. At present, constraint-based method is the only way to model and simulate a genome-scale metabolic network under steady-state criteria. Due to the inadequate assumption of the relationship in gene-enzyme-reaction as one-to-one unique association, computational difficulty or ignoring the yield from substrate to product, previous pathway finding approaches can’t be effectively applied to find out the high yield pathways that are mass balanced in stoichiometry. In addition, the shortest pathways may not be the pathways with high yield. At the same time, a pathway, which exists in stoichiometry, may not be feasible in thermodynamics. By using mixed integer programming strategy, we put forward an algorithm to identify all the smallest balanced pathways which convert the source compound to the target compound in large-scale metabolic networks. The resulting pathways by our method can finely satisfy the stoichiometric constraints and non-decomposability condition. Especially, the functions of high yield and thermodynamics feasibility have been considered in our approach. This tool is tailored to direct the metabolic engineering practice to enlarge the metabolic potentials of industrial strains by integrating the extensive metabolic network information built from systems biology dataset.

## Introduction

Metabolic network, the pseudo-steady state condition (PSSC): Genome-scale metabolic network (directed graph) is used to model the metabolism of biological systems, such as microorganisms. A few of models have been published including *E*. *coli* ^1^, *S*. *aureus* ^2^, *H*. *pylori* ^3^, *M*. *barkeri* ^4^, *S*. *cerevisiae* ^5^, *B*. *subtilis* ^6^, and so on. The pseudo-steady state condition (PSSC) refers to the main assumption that the concentration of internal compounds keeps invariable over time. Thus, internal compounds satisfy dx_c_/dt = 0 where x_c_ is the concentration of compound C^7^.

Source and target, external and internal compounds, exchange reactions: For a genome-scale metabolic network, exchange reactions are transport reactions through which cells exchange materials with the environment. External compounds are the compounds in the extracellular environment, but they enter the cell through exchange reactions and then play a rule. Source and target are respectively the start and the end of the pathways we hope to find.

Pathway and path: A metabolic pathway (a subset of the whole metabolic network) is a set of reactions by which a living organism transforms a source compound into a target compound^8^. Within a graph representation of a metabolic network, there may be multiple pathways. From the source compound to the target compound, there is a directed path with no cycles and in a particular determined metabolic pathway, and we refer to this directed path as metabolic path^9^. Of course and especially, when the pathway is branched, it may not be unique for this path. The metabolic pathway contains all the compounds and reactions involved in the pathway, all the internal compounds must be mass balanced in PSSC. Non-decomposability condition means that a pathway can’t be separated into two or more independent pathways.

Smallest pathway in large-scale metabolic networks: For a metabolic network, many pathways may have no biological meaning and if we can find experimentally determined pathways, this may provide in-depth knowledge for biomedical or biotechnological applications. So methodologies on metabolic pathway will devote to discover biologically meaningful metabolic pathways in metabolic networks. There may be many pathways between a source and a target in a large-scale network, and it would be computationally impracticable to completely enumerate all these pathways. Thus, pathway finding methods should focus on finding a set of pathways which were defined by the stoichiometric constraints and could be able to span the complete solution space of pathways. The smallest pathway is defined as the pathway with least reactions which convert the source compound to the target compound. Although pathway research should not come down only to the smallest pathway, the smallest pathway is an important aspect of biological meaning^10^.

Pathway finding and approaches: As we mentioned above, pathway finding approaches aim to find a set of pathways that should satisfy the stoichiometric constraints, so they can be called stoichiometric approaches. 1) Genetically independent pathways (GIP): Seressiotis and Bailey^11,12^ provided a method to discover a set of genetically independent pathways and their work represented the first stoichiometric methodology for the computation of metabolic pathways. But their algorithm required big computational effort, so could deal with only metabolic networks of relatively small size. In addition, their approach was based on the assumption that the relationship in gene-enzyme-reaction was a one-to-one unique association. 2) Improved genetically independent pathways (IGIP): Mavrovouniotis^13–15^ developed the algorithm of Seressiotis and Bailey, and used it to deal with pathways which comprised multiple targets and sources. His approach can be applied to a moderate size of the metabolic network. 3) Elementary flux modes: Elementary flux modes (EFMs), i.e. non-decomposable pathways at PSSC, were named by Schuster and co-workers^16^. With the increase in the size of the metabolic network, the number of EFMs entails combinatorial fashion^17^. In order to overcome this combinatorial explosion, different strategies have been adopted^18–20^. 4) Extreme pathways: Extreme pathways (EPs), a refined set of EFMs, were proposed by Schilling *et al*.^21^. Apart from the non-decomposability condition and the PSSC defined above, the systemic independence condition must be satisfied by the set of EPs, i.e. no EP can be written as a non-trivial nonnegative linear combination of other EPs^8^. As for EFMs, when applied to large-scale networks, computing all the EPs will suffer a combinatorial explosion. But enumerating special EFMs or EPs, such as from a substrate to a product in a given large-scale metabolic network, is computationally feasible. 5) k-shortest EFMs and flux paths: Figueiredo and Planes have presented a method to find the shortest elementary flux modes in genome-scale metabolic networks with integer programming^10^. By examining carefully the paper and doing computational practice, we found that this method did not consider ATP maintenance and the yield from substrate to product, and at the same time it did not provide the actual flux distribution in the identified pathways.

High yield and thermodynamics feasibility for a pathway: In the area of industrial biotechnology, improving bacterium is an important task and a high yield from substrate to product is the first target. For the construction of microorganism, we should utilize the pathway with high yield. The shortest EFMs may not be pathways with high yield and they are not equivalent to each other. But the two sets of pathways usually were regarded as equivalent, as stated in the literature^10^. We will show the difference and give a comparison in the result section of this paper. At the same time, although a pathway exists in stoichiometry, it may not always be feasible in thermodynamics. If we regard a pathway as an overall reaction and if we hope it is able to proceed spontaneously, it should satisfy the requirement of free energy change. Moreover, if there are several pathways which satisfy the condition, which one is more probable to occur in the cell?

Motivation and our contribution: At present, the modeling and simulation method for the genome-scale metabolic network is constraint-based method which satisfies steady-state criteria. For the reason of inadequacy assumption (GIP, IGIP), computational difficulty (EFMs, EPs), or ignoring the yield (k-shortest EFMs and flux paths), previous pathway finding approaches as we stated above can’t effectively design optimal pathways to direct the metabolic engineering practice. In this work, by using MIP (Mixed Integer Programming) strategy we put forward an algorithm to identify the smallest balanced pathways (SBPs) which convert the source compound to the target compound in large-scale metabolic networks. Under PSSC, the resulting SBPs of our method can well satisfy the stoichiometric constraints and non-decomposability condition; Multiple pathways which meet the above-mentioned criteria can be found and provided as candidate design; In addition, high yield is a new function; Especially, thermodynamics feasibility has been considered in our approach. The smallest pathways founded by our method can provide good references in the pathway design for the industrial microorganism. Our model can be easily solved by existing optimization software.

## Methods

### Mathematical description of metabolic network, Flux balance, and FBA

Usually, we can use a stoichiometric matrix, ***S***, to describe genome-scale metabolic network and the elements in ***S*** are the coefficients of reactions^22^. Under steady-state criteria, the time derivatives of metabolite concentrations are zero^7^, i.e. those internal metabolites should satisfy mass balance, so the equations of mass balance for all the metabolites can be represented as follows

1 $$S\cdot v={\bf{0}}$$

2 $${\alpha }_{i}\le {v}_{i}\le {\beta }_{i},\,i\in R$$

where ***S*** is the stoichiometric matrix, and *α*_*i*_ and *β*_*i*_ define the bounds through each reaction *v*_*i*_, R is the set of reactions.

As for metabolic networks in genome-scale, the fluxes within a cell usually can be computed with flux balance analysis (FBA) that can give optimal growth phenotypes, though not unique. In mathematics, FBA is an equivalent to a large-scale linear programming (LP). In our algorithm, we confine source and target compounds to be external compounds, i.e. there are exchange reactions related to them. For example, for the genome-scale metabolic network of *E*. *coli*_iJO1366^1^, there are more than 300 exchange reactions and we can choose any two as source and target.

### Mathematical model to find the smallest balanced pathway

In order to find the smallest balanced pathways in large-scale metabolic networks, MIP strategy is used as the mathematic model. We introduce binary variable *y* of the same number of continuous variable ***v*** to indicate the absence or presence of a reaction *v*_i_.

If *y*_i_ = 0 then *v*_i_ = 0 and If *y*_i_ = 1 then *α*_i_ ≤ *v*_i_ ≤ *β*_i_, we can express this idea as a constraint:

3 $${y}_{{\rm{i}}}\cdot {\alpha }_{{\rm{i}}}\le {v}_{{\rm{i}}}\le {y}_{{\rm{i}}}\cdot {\beta }_{{\rm{i}}},\,{y}_{{\rm{i}}}\in \{0,1\}\,{\rm{binary}}$$

The source and target nodes should be external nodes, and there are exchange reactions connected to them. In order to give a connected pathway, two bounds are added.

4 $${v}_{s}\le -{\rm{constant}}1;\,{v}_{t}\ge {\rm{constant}}2$$

The reason for the small of *v*_*t*_ is to let *v*_i_ of other reactions in the pathway not be beyond their constraints, and here constant1 and constant2 are positive values. Equation (4) is clearer in describing the input and output of the SBP than those methods of k-shortest EFMs^10^ and flux paths^23^.

Now we choose the sum of the number of used reactions as the objective function, i.e.

5 $${\rm{Obj}}=\sum {y}_{{\rm{i}}}$$

The strategy to find the smallest balanced pathways in large-scale metabolic networks may be expressed as a MIP model with *v*_i_ as continuous variable and *y*_i_ as a binary variable.

6a $$\mathrm{Minimize}:\,{\rm{Obj}}=\sum {{y}}_{{\rm{i}}}$$

6b $${\boldsymbol{S}}\cdot {\boldsymbol{v}}={\bf{0}}$$

6c $${\alpha }_{{\rm{i}}}\le {v}_{{\rm{i}}}\le {\beta }_{{\rm{i}}},\,{\rm{i}}\in {\rm{R}}$$

6d $${y}_{{\rm{i}}}\cdot {\alpha }_{{\rm{i}}}\le {v}_{{\rm{i}}}\le {y}_{{\rm{i}}}\cdot {\beta }_{{\rm{i}}}$$

6e $${y}_{i}\in \{0,1\}\,{\rm{binary}}$$

6f $${v}_{s}\le -{\rm{constant}}1,\,{{v}}_{t}\ge {\rm{constant}}2$$

The SBPs is different from the null space of the stoichiometric matrix and the null space of the stoichiometric matrix is only the constraints (1). The SBPs is smaller than the null space of the stoichiometric matrix.

### Extend to custom-specified conditions

For this model, we can easily preset the metabolic network to meet the requirement of the specific situations. For example, certain reactions must not be appearing, or some genes are to be disrupted, we just preset v_i_ = 0; In other case, certain reactions must be reversible, we can preset v_min_ = −1000, v_max_ = 1000. These could be achieved by setting the boundaries of the reactions. Then the solution of smallest balanced pathways is within the scope of the given conditions.

### Solve the model and obtain multi solutions

For MIP, some existing software can be used to find its solution and we use Gurobi^24^ here. With a statistic of the fluxes which are not zero in absolute value (or larger than a given small value 10^−5^) or which *y*_i_ is 1 (the two ways are consistent), we can determine those reactions which should appear, and further, we can obtain the smallest balanced pathways.

Sometimes, there exist different states of integer variables but the objective value is the same, i.e. a MIP may have multi integer solutions. Up to date, as we know, there does not exist optimization tool which can give directly multi integer solutions for a MIP. Here we utilize a method proposed by Balas and Jeroslow, named Combinatorial Bender’s cut^25^. The approach of Bender’s cut is that iteration is used from an existing solution, at the same time in each iteration to exclude an existing solution by adding the following binary cut

7 $$\sum _{i\in B}{y}_{i}-\sum _{i\in N}{y}_{i}\le |B|-1,\,B=\{i|{y}_{i}=1\},\,N=\{i|{y}_{i}=0\}$$

All the multi integer solutions will be got by this way.

### Smallest balanced pathway with high yield

SBPs have the least number of reactions but may not have the high yield of a chemical which the microorganism produces. High yield means a high amount of desired product and little or no by-product which might make the downstream complicated, costly, and polluted. In another word, high yield means cost-saving. Sometimes, high yield is our interesting aspect, so it is best to find SBPs with high yield. In order to estimate the reachable high yield of the chemical, we can use FBA with the exchange reaction rate v_chem_ of this chemical as the objective and we will get the theoretical ratio V_max_. Then we can use 95% of the value of highest yield V_max_ as a constraint in our MIP model. Finally, we will get all SBPs with a high yield which is larger than 95% of the value of highest yield.

8 $${{\rm{v}}}_{{\rm{chem}}}\ge 0.95\times {{\rm{V}}}_{{\rm{\max }}}$$

### Thermodynamics feasibility analysis

Although a pathway exists in stoichiometry, it may not always be feasible in thermodynamics. If we regard a pathway as an overall reaction and if we hope it is able to proceed spontaneously, it should satisfy the requirement that each reaction in the pathway must be thermodynamically feasible individually, i.e. the flux and the free energy change of this reaction must have opposite signs or the reaction is reversible. The data of free energy change for a microbe is not rich in literature and the first one is *E*. *coli* ^26,27^. The thermodynamic data of *E*. *coli* model was calculated by Group Contribution Method^27,28^. There is a range of free energy change for every reaction and it is calculated by min/max delta G. The range of delta G could be used to decide the reversibility of a reaction.

## Results

### Case 1: The SBPs from glucose to succinic acid in given conditions

#### The SBPs from glucose to succinic acid

In this example, we hope to know how succinic acid is synthesized by glucose with *E*. *coli*. By using our algorithm, we computed out the smallest balanced pathways from glucose (source compound, input exchange reaction is EX_glc[e]) to succinic acid (target compound, output exchange reaction is EX_succ[e]) in the genome-scale metabolic network of *E*. *coli* (its SBML model is iJO1366^1^). In the process of computation, we restrict the input rate of glucose (v_s_ = −100 mmol/g(Dw)h) and the output rate of succinic acid (v_t_ ≥ 0.01 mmol/g(Dw)h). The given conditions are that we restrict the input and output of the cell to be only five compounds, i.e. glucose, succinic acid, CO_2_, H_2_O, and H. The reason for restricting only these five compounds is that succinic acid can be synthesized by them. There are 12 alternative solutions for this model, seeing Supplementary Material a, and all the solutions have 37 step reactions. Among 12 alternative solutions, 31 step reactions are the same, and they are “ACONTa, ACONTb, ATPM, ATPS4rpp, CO2tex, CO2tpp, CS, ENO, EX_co2(e), EX_glc(e), EX_h2o(e), EX_h(e), EX_succ(e), FBA, FUM, GAPD, GLCt2pp, H2Otex, H2Otpp, Htex, ICL, MALS, MDH, PDH, PFK, PGK, PGM, PPC, SUCCt3pp, SUCCtex, TPI”, while 11 step reactions are different, illustrated in Table 1. The whole names of each reaction in these 12 pathways are provided in the Supplementary Material a.

**Table 1:** 11 reactions which are different among 12 alternative solutions.

1	FRD3	GLCptspp	GLCtexi	HEX1	NADH18pp	PGI
2	FRD3	GLCptspp	GLCtex	HEX1	NADH18pp	PGI
3	FRD3	GLCtex	HEX1	NADH18pp	PGI	PYK
4	FRD3	GLCtex	HEX7	NADH18pp	PYK	XYLI2
5	FRD2	GLCtexi	HEX1	NADH17pp	PGI	PYK
6	FRD2	GLCtex	HEX7	NADH17pp	PYK	XYLI2
7	FRD2	GLCptspp	GLCtexi	HEX1	NADH17pp	PGI
8	FRD2	GLCtex	HEX1	NADH17pp	PGI	PYK
9	FRD2	GLCtexi	HEX7	NADH17pp	PYK	XYLI2
10	FRD3	GLCtexi	HEX7	NADH18pp	PYK	XYLI2
11	FRD2	GLCptspp	GLCtex	HEX1	NADH17pp	PGI
12	FRD3	GLCtexi	HEX1	NADH18pp	PGI	PYK

**Figure 1:**
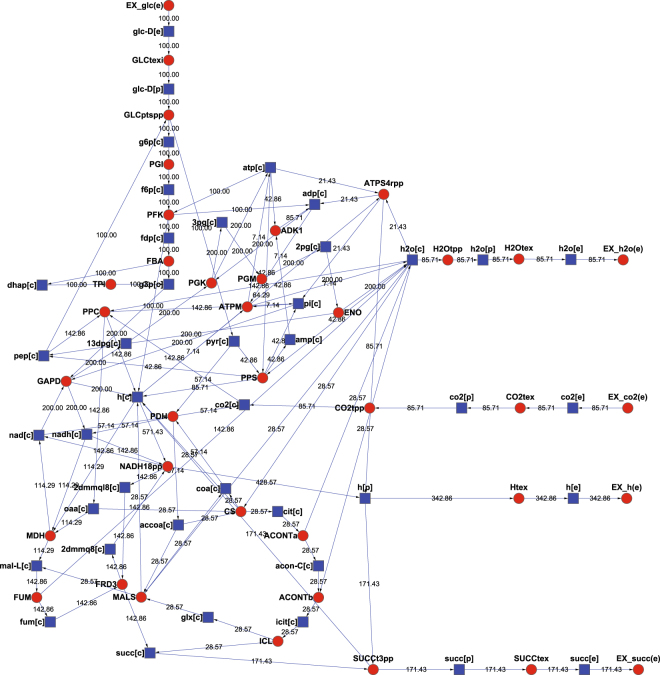
One of the smallest balanced pathways from glucose to succinic acid in the genome-scale metabolic network of *E*. *coli* under given conditions, including 37 reactions and 41 compounds. The number marked beside every line represents consuming or producing rate of compounds.

One of these pathways, the first solution, was illustrated in Fig. 1 with red and circle nodes for reactions and with blue and square nodes for compounds. The pathway included 37 reactions and 41 compounds. The number marked beside each line represents the rate of consuming or producing the corresponding compound. For every compound, its mass is balanced, i.e. the sum rate consuming it is equal to the sum rate producing it. At the same time, this pathway includes the least reactions among all the pathways converting glucose to succinic acid.

From this pathway, we can know clearly the pathway to synthesize succinic acid and the balanced proportions among fluxes through every reaction in this pathway. We know also how these enzymes (or reactions) cooperate with each other to synthesize succinic acid. This means these reactions are equally important to fulfill the overall function of succinic acid production. The pathways including the reactions, genes, and fluxes should be regarded as ideal references to guide strain engineering activity. This will greatly reduce the scope of targets to enhance genes in order to accelerate the speed of producing succinic acid.

#### Thermodynamics feasibility analysis

With the data of free energy change of each reaction for *E*. *coli* ^26,27^, seeing Supplementary Material a, we have made a statistic on the free energy change delta G and the range of delta G of individual reactions for each of the above 12 alternative pathways producing succinic acid, illustrated in Table 2. For each pathway, the fluxes and their corresponding free energy changes of these reactions either have opposite signs or the reactions are reversible, the number of irreversible reactions that the fluxes and their corresponding free energy changes have the same signs is zero, and so all these pathways are feasible in thermodynamics.

**Table 2:** Statistic on the thermodynamic data for each of the above 12 alternative pathways.

No. of pathway	Patheway-1	Patheway-2	Patheway-3	Patheway-4	Patheway-5	Patheway-6
Nos	16	16	16	16	16	16
Nzo	10	10	10	10	10	10
Nssr	6	6	6	6	6	6
Nssi	0	0	0	0	0	0
Nex	5	5	5	5	5	5
Total	37	37	37	37	37	37
**No. of pathway**	**Patheway-7**	**Patheway-8**	**Patheway-9**	**Patheway-10**	**Patheway-11**	**Patheway-12**
Nos	16	16	16	16	16	16
Nzo	10	10	10	10	10	10
Nssr	6	6	6	6	6	6
Nssi	0	0	0	0	0	0
Nex	5	5	5	5	5	5
Total	37	37	37	37	37	37

Nos: number of reactions that the fluxes and their corresponding free energy changes have opposite signs.

Nzo: number of reactions that the free energy changes are zero.

Nssr: number of reversible reactions that the fluxes and their corresponding free energy changes have the same signs.

Nssi: number of irreversible reactions that the fluxes and their corresponding free energy changes have the same signs.

Nex: number of exchange reactions.

#### Comparison between SBPs with high yield and those without high yield

In the above succinic acid case, the yield 171.43:100 has almost been the theoretical value. In order to show the difference of those with high yield and SBPs without high yield, threonine production with *E*. *coli* is selected for the study. Threonine is an important chemical in industry, which can be produced by *E*. *coli* with glucose. We have computed all the threonine SBPs with highest yield in *E*. *coli* with glucose as substrate, and get 16 SBPs. All the SBPs have 50 step-reactions and the yield is 1.248:1 in a molar ratio which is near the theoretical yield. All the SBPs are in Supplementary Material b. At the same time, we cancel the high yield function of our algorithm and run our algorithm again. Now there are 7 SBPs and all the SBPs have 43 steps of reactions, but the yield is only 0.25:1 in molar ratio. So although the steps are less, the yield is smaller than that of SBPs with high yield. All the SBPs without high yield are in Supplementary Material c. Of course, in the practice of synthesizing threonine, the SBPs with high yield has more significance for commercialization.

#### Comparison with k-shortest EFMs

In order to make a quantitative comparison with conventional methods, the method of k-shortest EFMs was selected as it is the nearest approach to ours. We use this algorithm to compute out all the shortest EFMs from glucose to succinate in the genome-scale metabolic network of *E*. *coli*_iJO1366, illustrated in Supplementary Material d. All the 24 shortest EFMs are 30 step reactions, they are shorter than our above SBPs from glucose to succinate, but the molar yields of these EFMs are 1.0, while the molar yields of our SBPs are 1.71, which is near the theoretical ratio. We checked these EFMs and found the main reason was that they did not consider ATP maintenance and the maximum conversion yield. The SBPs with high yield will be more helpful in the practices of synthesizing chemicals with microbes. ATP maintenance, i.e. ATPM reaction, an artificial reaction, is necessary for the cell to maintain the physiological behaviors of microbes. If we reject ATP maintenance and the requirement of high yield, our SBP algorithm will get a similar result of shortest EFMs. Another aspect is the approach of shortest EFMs did not provide the flux distribution in the computed pathway and all the reaction flux is 1, while our SBP algorithm can give the actual flux distribution in the computed pathway. Flux distribution is a fine reference in pathway design when we want to synthesize chemicals with microbes.

### Case 2: The SBPs from glucose to a variety of chemicals which *E. coli* can produce with maximum productivity

In addition to succinic acid and threonine that we mentioned above, *E*. *coli* can produce many other chemicals such as lactic acid, formic acid, fumaric acid and so on. In the model iJO1366, there are 324 exchange reactions. Only 25 reactions have low bounds which less than 0, while all the up bounds equal to 1000. We use glucose (start or source) as the input, at the same time use all these 324 reactions except for glucose as the output (target) respectively, and calculate the SBPs with maximum productivity for every chemical. For a given chemical, if *E*. *coli* can’t produce it, i.e. the maximum productivity for it is zero, the algorithm will not return its SBPs.

**Figure 2:**
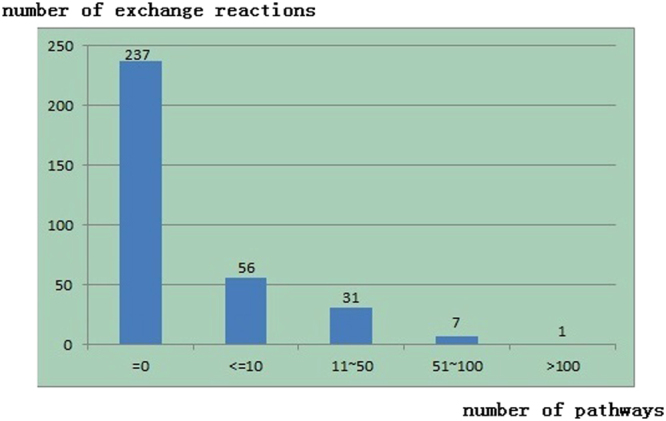
Statistics for all the SBPs to a variety of chemicals which *E*. *coli* can produce.

We have made a statistics for all the SBPs to a variety of chemicals which *E*. *coli* can produce and found that in many cases, the number of SBPs is less than 10 and that those cases which are larger than 100 only take a very small proportion, as shown in Fig. 2. Here, we do not provide thermodynamics feasibility analysis (TFA) for each SBP. If we have interest for a certain SBPs in their thermodynamics feasibility, we can do TFA by the method we provided in the section of Methods.

## Conclusions

### Main idea and difference from previous algorithms

Up to date, modeling a genome-scale metabolic network in dynamics is still beyond the access of most laboratories, so the best way to make use of flooding metabolic network information to direct the metabolic engineering practice is the constraint-based approach which satisfies the pseudo-steady state condition (PSSC). A pathway that converts a given source compound to a given target compound should satisfy the stoichiometric constraints and non-decomposability condition. EFMs and EPs are two pathway finding approaches, but calculating the set of EPs or enumerating all the EFMs will suffer a combinatorial explosion when applied to large networks. Existed approaches of k-shortest EFMs and flux paths are not the methods of considering the yield from substrate to product which is highly biotechnologically relevant.

In this work, by utilizing Mixed Integer Programming (MIP), we present an approach for pathway finding. Our algorithm has a number of good features: 1) It is a method of thorough stoichiometry. It can identify the balanced pathways in the genome-scale metabolic network. The balance here means that the mass of internal compounds is balanced, i.e. stoichiometric balance. The smallest means that the pathway identified has least reactions. 2) The pathways found by this approach are usually short enough, which simplify the metabolic engineering practice and also save the cellular energy consumption for synthesizing proteins for the reactions. It is well-known that protein synthesis is the most energy-intensive process. 3) Our algorithm can return all the alternative solutions, and this can provides more choices in industrial stain design. 4) High yield can be added as required condition, which is important for biotechnology purpose. 5) Thermodynamics data are integrated to allow the thermodynamics feasibility analysis.

We recognized that although the short pathway has the advantages as mentioned above, the shortest pathway may not necessarily be biologically feasible and some biological pathways are not the shortest one in nature. Our algorithm is to break the evolutionary barrier and eventually help to create artificial cell factory. Furthermore, by just simply modifying our code, we can easily found all balanced pathway with the length of shortest plus 1, 2, …, and so on.

### Computational complexity

The model of our approach comes down to a MIP and MIP is an essentially combinational problem. Computational complexity will be proportional to the scale of the problem. But for a large scale problem, existing solving software can solve it in not long time. Such as our case study with several thousand of variables, the computation time will take just several minutes by an HPC (high-performance computer) with 48 cores.

### Application of industrial stain design

In industrial stain design, high yield from the source substrate to the target product is the first important aspect. To implement this, biologically feasible and high yield pathways should be utilized. The smallest balanced pathway with high yield can provide an ideal reference to guide metabolic engineering practice. In particular, the balanced pathways tells many co-dancing reactions which are beyond the sight of normal biological knowledge. The metabolic bottleneck may not necessarily locate on the traditional biochemical pathway. The accessory reaction which is responsible to recycle the cofactors, intermediate or to supply the precursors is shown to be as important as the reactions in the known biochemical pathway. Meanwhile, the relative strength of the fluxes of different reactions also tells the metabolic engineer how to fine-tune the relative activities of different reactions. Integrating with the experimental determination of intracellular transcriptome, proteome and even metabolome, metabolic engineering should be able to identify the potential rate-limiting reactions which they need to put effort on.

In our first case study, producing succinic acid with *E*. *coli*, the input rate of glucose is 100 mmol/g(Dw)h and the output rate of succinic acid is 171 mmol/g(Dw)h, so this pathway almost reaches the theoretical ratio of glucose/succinic acid in *E*. *coli*. If we fulfill the pathway in *E*. *coli*, it can make a good utilization of glucose in producing succinic acid. SBPs with high yield from glucose to a variety of chemicals, which *E*. *coli* can produce, have been calculated by our algorithm. Especially, we can make decisions on the thermodynamics feasibility by integrating the data of free energy change.

## Electronic supplementary material

Supplementary material a

Supplementary material b/c/d
