# Supplementary material for: Find_tfSBP: find thermodynamics-feasible and smallest balanced pathways with high yield from large-scale metabolic networks
Source: Sci Rep. 2017 Dec 11;7:17334. doi: 10.1038/s41598-017-17552-2 (PMC5725421; doi:10.1038/s41598-017-17552-2)
Supplement: Supplementary file 2 — Supplementary material b/c/d [file 41598_2017_17552_MOESM2_ESM.pdf]

Find_tfSBP: find thermodynamics-feasible and smallest balanced pathways with high yield from large-scale metabolic networks

Zixiang Xu[1 2 §] Jibin Sun[2 §] Qiaqing Wu[1] Dunming Zhu[1]

1 National Engineering Laboratory for Industrial Enzymes and Tianjin Engineering Center for Biocatalytic Technology, Tianjin Institute of Industrial Biotechnology, Chinese Academy of Sciences, Tianjin 300308, China

2 Key laboratory of systems microbial biotechnology, Tianjin Institute of Industrial Biotechnology, Chinese Academy of Sciences, Tianjin 300308, China

Supplementary material b

16 SBPs with highest conversion rate in *E. coli* from glucose to threonine.

1. 'EX_co2(e), EX_glc(e), EX_h(e), EX_h2o(e), EX_nh4(e), EX_o2(e), EX_thr-L(e), ASAD, ASPK, ASPTA, ATPM, ATPS4rpp, CO2tex, CO2tpp, CYTBO3_4pp, ENO, FBA3, G6PDH2r, GAPD, GLCabcpp, GLCtexi, GLUDy, GND, H2Otex, H2Otpp, HEX1, HSDy, HSK, Htex, NADH16pp, NADTRHD, NH4tex, NH4tpp, O2tex, O2tpp, PFK_3, PGI, PGK, PGL, PGM, PPC, RPE, RPI, TALA, THRS, THRt2rpp, THRtex, TKT1, TKT2, TPI'

2. 'EX_co2(e), EX_glc(e), EX_h(e), EX_h2o(e), EX_nh4(e), EX_o2(e), EX_thr-L(e), ASAD, ASPK, ASPTA, ATPM, ATPS4rpp, CO2tex, CO2tpp, CYTBO3_4pp, ENO, FBA, G6PDH2r, GAPD, GLCabcpp, GLCtex, GLUDy, GND, H2Otex, H2Otpp, HEX1, HSDy, HSK, Htex, NADH16pp, NADTRHD, NH4tex, NH4tpp, O2tex, O2tpp, PFK, PGI, PGK, PGL, PGM, PPC, RPE, RPI, TALA, THRS, THRt2rpp, THRtex, TKT1, TKT2, TPI'

3. 'EX_co2(e), EX_glc(e), EX_h(e), EX_h2o(e), EX_nh4(e), EX_o2(e), EX_thr-L(e), ASAD, ASPK, ASPTA, ATPM, ATPS4rpp, CO2tex, CO2tpp, CYTBO3_4pp, ENO, FBA, G6PDH2r, GAPD, GLCt2pp, GLCtexi, GLUDy, GND, H2Otex, H2Otpp, HEX1, HSDy, HSK, Htex, NADH16pp, NADTRHD, NH4tex, NH4tpp, O2tex, O2tpp, PFK, PGI, PGK, PGL, PGM, PPC, RPE, RPI, TALA, THRS, THRt2rpp, THRtex, TKT1, TKT2, TPI'

4. 'EX_co2(e), EX_glc(e), EX_h(e), EX_h2o(e), EX_nh4(e), EX_o2(e), EX_thr-L(e), ASAD, ASPK, ASPTA, ATPM, ATPS4rpp, CO2tex, CO2tpp, CYTBO3_4pp, ENO, FBA3, G6PDH2r, GAPD, GLCt2pp, GLCtexi, GLUDy, GND, H2Otex, H2Otpp, HEX1, HSDy, HSK, Htex, NADH16pp, NADTRHD, NH4tex, NH4tpp, O2tex, O2tpp, PFK_3, PGI,

PGK, PGL, PGM, PPC, RPE, RPI, TALA, THRS, THRt2rpp, THRtex, TKT1, TKT2, TPI'

5. 'EX_co2(e), EX_glc(e), EX_h(e), EX_h2o(e), EX_nh4(e), EX_o2(e), EX_thr-L(e), ASAD, ASPK, ASPTA, ATPM, ATPS4rpp, CO2tex, CO2tpp, CYTBO3_4pp, ENO, FBA, G6PDH2r, GAPD, GLCt2pp, GLCtexi, GLUDy, GND, H2Otex, H2Otpp, HEX1, HSDy, HSK, Htex, NADH16pp, NADPHQR2, NH4tex, NH4tpp, O2tex, O2tpp, PFK, PGI, PGK, PGL, PGM, PPC, RPE, RPI, TALA, THRS, THRt2rpp, THRtex, TKT1, TKT2, TPI'

6. 'EX_co2(e), EX_glc(e), EX_h(e), EX_h2o(e), EX_nh4(e), EX_o2(e), EX_thr-L(e), ASAD, ASPK, ASPTA, ATPM, ATPS4rpp, CO2tex, CO2tpp, CYTBO3_4pp, ENO, FBA, G6PDH2r, GAPD, GLCt2pp, GLCtex, GLUDy, GND, H2Otex, H2Otpp, HEX1, HSDy, HSK, Htex, NADH16pp, NADTRHD, NH4tex, NH4tpp, O2tex, O2tpp, PFK, PGI, PGK, PGL, PGM, PPC, RPE, RPI, TALA, THRS, THRt2rpp, THRtex, TKT1, TKT2, TPI'

7. 'EX_co2(e), EX_glc(e), EX_h(e), EX_h2o(e), EX_nh4(e), EX_o2(e), EX_thr-L(e), ASAD, ASPK, ASPTA, ATPM, ATPS4rpp, CO2tex, CO2tpp, CYTBO3_4pp, ENO, FBA, G6PDH2r, GAPD, GLCt2pp, GLCtex, GLUDy, GND, H2Otex, H2Otpp, HEX1, HSDy, HSK, Htex, NADH16pp, NADPHQR2, NH4tex, NH4tpp, O2tex, O2tpp, PFK, PGI, PGK, PGL, PGM, PPC, RPE, RPI, TALA, THRS, THRt2rpp, THRtex, TKT1, TKT2, TPI'

8. 'EX_co2(e), EX_glc(e), EX_h(e), EX_h2o(e), EX_nh4(e), EX_o2(e), EX_thr-L(e), ASAD, ASPK, ASPTA, ATPM, ATPS4rpp, CO2tex, CO2tpp, CYTBO3_4pp, ENO, FBA, G6PDH2r, GAPD, GLCabcpp, GLCtexi, GLUDy, GND, H2Otex, H2Otpp, HEX1, HSDy, HSK, Htex, NADH16pp, NADTRHD, NH4tex, NH4tpp, O2tex, O2tpp, PFK, PGI, PGK, PGL, PGM, PPC, RPE, RPI, TALA, THRS, THRt2rpp, THRtex, TKT1, TKT2, TPI'

9. 'EX_co2(e), EX_glc(e), EX_h(e), EX_h2o(e), EX_nh4(e), EX_o2(e), EX_thr-L(e), ASAD, ASPK, ASPTA, ATPM, ATPS4rpp, CO2tex, CO2tpp, CYTBO3_4pp, ENO, FBA, G6PDH2r, GAPD, GLCt2pp, GLCtexi, GLUDy, GND, H2Otex, H2Otpp, HEX1, HSDy, HSK, Htex, NADH16pp, NADTRHD, NH4tex, NH4tpp, O2tex, O2tpp, PFK, PGI, PGK, PGL, PGM, PPC, RPE, RPI, TALA, THRS, THRt2pp, THRtex, TKT1, TKT2, TPI'

10. 'EX_co2(e), EX_glc(e), EX_h(e), EX_h2o(e), EX_nh4(e), EX_o2(e), EX_thr-L(e), ASAD, ASPK, ASPTA, ATPM, ATPS4rpp, CO2tex, CO2tpp, CYTBO3_4pp, ENO, FBA3, G6PDH2r, GAPD, GLCt2pp, GLCtex, GLUDy, GND, H2Otex, H2Otpp, HEX1, HSDy, HSK, Htex, NADH16pp, NADPHQR2, NH4tex, NH4tpp, O2tex, O2tpp, PFK_3, PGI, PGK, PGL, PGM, PPC, RPE, RPI, TALA, THRS, THRt2rpp, THRtex, TKT1, TKT2, TPI'

11. 'EX_co2(e), EX_glc(e), EX_h(e), EX_h2o(e), EX_nh4(e), EX_o2(e), EX_thr-L(e), ASAD, ASPK, ASPTA, ATPM, ATPS4rpp, CO2tex, CO2tpp, CYTBO3_4pp, ENO, FBA3, G6PDH2r, GAPD, GLCt2pp, GLCtex, GLUDy, GND, H2Otex, H2Otpp, HEX1, HSDy, HSK, Htex, NADH16pp, NADTRHD, NH4tex, NH4tpp, O2tex, O2tpp, PFK_3, PGI, PGK, PGL, PGM, PPC, RPE, RPI, TALA, THRS, THRt2rpp, THRtex, TKT1, TKT2, TPI'

12. 'EX_co2(e), EX_glc(e), EX_h(e), EX_h2o(e), EX_nh4(e), EX_o2(e), EX_thr-L(e), ASAD, ASPK, ASPTA, ATPM, ATPS4rpp, CO2tex, CO2tpp, CYTBO3_4pp, ENO, FBA3, G6PDH2r, GAPD, GLCt2pp, GLCtexi, GLUDy, GND, H2Otex, H2Otpp, HEX1, HSDy, HSK, Htex, NADH16pp, NADPHQR2, NH4tex, NH4tpp, O2tex, O2tpp, PFK_3, PGI, PGK, PGL, PGM, PPC, RPE, RPI, TALA, THRS, THRt2rpp, THRtex, TKT1, TKT2, TPI'

13. 'EX_co2(e), EX_glc(e), EX_h(e), EX_h2o(e), EX_nh4(e), EX_o2(e), EX_thr-L(e), ASAD, ASPK, ASPTA, ATPM, ATPS4rpp, CO2tex, CO2tpp, CYTBO3_4pp, ENO, FBA, G6PDH2r, GAPD, GLCt2pp, GLCtex, GLUDy, GND, H2Otex, H2Otpp, HEX1, HSDy, HSK, Htex, NADH16pp, NADTRHD, NH4tex, NH4tpp, O2tex, O2tpp, PFK, PGI, PGK, PGL, PGM, PPC, RPE, RPI, TALA, THRS, THRt2pp, THRtex, TKT1, TKT2, TPI'

14. 'EX_co2(e), EX_glc(e), EX_h(e), EX_h2o(e), EX_nh4(e), EX_o2(e), EX_thr-L(e), ASAD, ASPK, ASPTA, ATPM, ATPS4rpp, CO2tex, CO2tpp, CYTBO3_4pp, ENO, FBA3, G6PDH2r, GAPD, GLCt2pp, GLCtexi, GLUDy, GND, H2Otex, H2Otpp, HEX1, HSDy, HSK, Htex, NADH16pp, NADTRHD, NH4tex, NH4tpp, O2tex, O2tpp, PFK_3, PGI, PGK, PGL, PGM, PPC, RPE, RPI, TALA, THRS, THRt2pp, THRtex, TKT1, TKT2, TPI'

15. 'EX_co2(e), EX_glc(e), EX_h(e), EX_h2o(e), EX_nh4(e), EX_o2(e), EX_thr-L(e), ASAD, ASPK, ASPTA, ATPM, ATPS4rpp, CO2tex, CO2tpp, CYTBO3_4pp, ENO, FBA3, G6PDH2r, GAPD, GLCt2pp, GLCtex, GLUDy, GND, H2Otex, H2Otpp, HEX1, HSDy, HSK, Htex, NADH16pp, NADTRHD, NH4tex, NH4tpp, O2tex, O2tpp, PFK_3, PGI, PGK, PGL, PGM, PPC, RPE, RPI, TALA, THRS, THRt2pp, THRtex, TKT1, TKT2, TPI'

16. 'EX_co2(e), EX_glc(e), EX_h(e), EX_h2o(e), EX_nh4(e), EX_o2(e), EX_thr-L(e), ASAD, ASPK, ASPTA, ATPM, ATPS4rpp, CO2tex, CO2tpp, CYTBO3_4pp, ENO, FBA3, G6PDH2r, GAPD, GLCabcpp, GLCtex, GLUDy, GND, H2Otex, H2Otpp, HEX1, HSDy, HSK, Htex, NADH16pp, NADTRHD, NH4tex, NH4tpp, O2tex, O2tpp, PFK_3, PGI, PGK, PGL, PGM, PPC, RPE, RPI, TALA, THRS, THRt2rpp, THRtex, TKT1, TKT2, TPI'

Supplementary material c

7 SBPs without highest conversion rate in *E. coli* from glucose to threonine.

1. 'EX_ac(e), EX_glc(e), EX_h(e), EX_h2o(e), EX_lac-D(e), EX_nh4(e), EX_thr-L(e), ACKr, ACt2rpp, ACtex, ASAD, ASPK, ASPTA, ATPM, D-LACt2pp, D-LACtex, ENO, FBA, GAPD, GLCptspp, GLCtexi, GLUDy, H2Otex, H2Otpp, HSDy, HSK, Htex, LDH_D, MDH, ME2, NH4tex, NH4tpp, PDH, PFK, PGI, PGK, PGM, PPC, PTAr, THRS, THRt2rpp, THRtex, TPI'

2. 'EX_ac(e), EX_glc(e), EX_h(e), EX_h2o(e), EX_lac-D(e), EX_nh4(e), EX_thr-L(e), ACKr, ACt2rpp, ACtex, ASAD, ASPK, ASPTA, ATPM, D-LACt2pp, D-LACtex, ENO, FBA, GAPD, GLCptspp, GLCtex, GLUDy, H2Otex, H2Otpp, HSDy, HSK, Htex, LDH_D, MDH, ME2, NH4tex, NH4tpp, PDH, PFK, PGI, PGK, PGM, PPC, PTAr, THRS, THRt2rpp, THRtex, TPI'

3. 'EX_co2(e), EX_glc(e), EX_h(e), EX_h2o(e), EX_lac-D(e), EX_nh4(e), EX_pyr(e), EX_thr-L(e), ASAD, ASPK, ASPTA, ATPM, CO2tex, CO2tpp, D-LACt2pp, D-LACtex, ENO, FBA, GAPD, GLCptspp, GLCtexi, GLUDy, H2Otex, H2Otpp, HSDy, HSK, Htex, LDH_D, MDH, ME2, NH4tex, NH4tpp, PFK, PGI, PGK, PGM, PPC, PYRt2rpp, PYRtex, THRS, THRt2rpp, THRtex, TPI'

4. 'EX_co2(e), EX_glc(e), EX_h(e), EX_h2o(e), EX_lac-D(e), EX_nh4(e), EX_pyr(e), EX_thr-L(e), ASAD, ASPK, ASPTA, ATPM, CO2tex, CO2tpp, D-LACt2pp, D-LACtex, ENO, FBA, GAPD, GLCptspp, GLCtex, GLUDy, H2Otex, H2Otpp, HSDy, HSK, Htex, LDH_D, MDH, ME2, NH4tex, NH4tpp, PFK, PGI, PGK, PGM, PPC, PYRt2rpp, PYRtex, THRS, THRt2rpp, THRtex, TPI'

5. 'EX_ac(e), EX_glc(e), EX_h(e), EX_h2o(e), EX_lac-D(e), EX_nh4(e), EX_thr-L(e), ACALD, ACt2rpp, ACtex, ALDD2y, ASAD, ASPK, ASPTA, ATPM, D-LACt2pp, D-LACtex, ENO, FBA, GAPD, GLCptspp, GLCtexi, GLUDy, H2Otex, H2Otpp, HSDy, HSK, Htex, LDH_D, MDH, ME2, NH4tex, NH4tpp, PDH, PFK, PGI, PGK, PGM, PPC, THRS, THRt2rpp, THRtex, TPI'

6. 'EX_ac(e), EX_glc(e), EX_h(e), EX_h2o(e), EX_lac-D(e), EX_nh4(e), EX_thr-L(e), ACALD, ACt2rpp, ACtex, ALDD2y, ASAD, ASPK, ASPTA, ATPM, D-LACt2pp, D-LACtex, ENO, FBA, GAPD, GLCptspp, GLCtex, GLUDy, H2Otex, H2Otpp, HSDy, HSK, Htex, LDH_D, MDH, ME2, NH4tex, NH4tpp, PDH, PFK, PGI, PGK, PGM, PPC, THRS,

THRt2rpp, THRtex, TPI'

7. 'EX_ac(e), EX_glc(e), EX_h(e), EX_h2o(e), EX_lac-D(e), EX_nh4(e), EX_thr-L(e), ACt2rpp, ACtex, ASAD, ASPK, ASPTA, ATPM, CITL, CS, D-LACt2pp, D-LACtex, ENO, FBA, GAPD, GLCptspp, GLCtexi, GLUDy, H2Otex, H2Otpp, HSDy, HSK, Htex, LDH_D, MDH, ME2, NH4tex, NH4tpp, PDH, PFK, PGI, PGK, PGM, PPC, THRS, THRt2rpp, THRtex, TPI'

Supplementary material d

24 EFMs from glucose to succinic acid in E.coli_iJO1366

1, EX_acald(e), EX_glc(e), EX_h(e), EX_h2o(e), EX_succ(e), ACALD, ACALDtex, ACALDtpp, DHAPT, ENO, F6PA, FRD3, FUM, GAPD, GLCabcpp, GLCtexi, H2Otex, H2Otpp, HEX1, Htex, MDH, NADH18pp, PDH, PGI, PGK, PGM, PPC, SUCCt3pp, SUCCtex, TPI

2, EX_acald(e), EX_glc(e), EX_h(e), EX_h2o(e), EX_succ(e), ACALD, ACALDtex, ACALDtpp, DHAPT, ENO, F6PA, FRD3, FUM, GAPD, GLCabcpp, GLCtex, H2Otex, H2Otpp, HEX1, Htex, MDH, NADH18pp, PDH, PGI, PGK, PGM, PPC, SUCCt3pp, SUCCtex, TPI

3, EX_acald(e), EX_glc(e), EX_h(e), EX_h2o(e), EX_succ(e), ACALD, ACALDtex, ACALDtpp, ENO, FBA, FBP, FRD3, FUM, GAPD, GLCptspp, GLCtexi, H2Otex, H2Otpp, Htex, MDH, NADH18pp, PDH, PFK, PGI, PGK, PGM, PPC, SUCCt3pp, SUCCtex, TPI

4, EX_acald(e), EX_glc(e), EX_h(e), EX_h2o(e), EX_succ(e), ACALD, ACALDtex, ACALDtpp, ENO, FBA, FRD2, FUM, GAPD, GLCptspp, GLCtex, H2Otex, H2Otpp, Htex, MDH, NADH17pp, PDH, PFK, PGI, PGK, PGM, PPC, PPCK, SUCCt3pp, SUCCtex, TPI

5, EX_acald(e), EX_glc(e), EX_h(e), EX_h2o(e), EX_succ(e), ACALD, ACALDtex, ACALDtpp, DHAPT, ENO, F6PA, FRD2, FUM, GAPD, GLCabcpp, GLCtexi, H2Otex, H2Otpp, HEX7, Htex, MDH, NADH17pp, PDH, PGK, PGM, PPC, SUCCt3pp, SUCCtex, TPI, XYLI2

6, EX_acald(e), EX_glc(e), EX_h(e), EX_h2o(e), EX_succ(e), ACALD, ACALDtex, ACALDtpp, DHAPT, ENO, F6PA, FRD3, FUM, GAPD, GLCabcpp, GLCtex, H2Otex, H2Otpp, HEX7, Htex, MDH, NADH18pp, PDH, PGK, PGM, PPC, SUCCt3pp, SUCCtex, TPI, XYLI2

7, EX_acald(e), EX_glc(e), EX_h(e), EX_h2o(e), EX_succ(e), ACALD, ACALDtex, ACALDtpp, ATPM, ENO, FBA, FRD2, FUM, GAPD, GLCptspp, GLCtexi, H2Otex, H2Otpp, Htex, MDH, NADH17pp, PDH, PFK, PGI, PGK, PGM, PPC, SUCCt3pp, SUCCtex, TPI

8, EX_acald(e), EX_glc(e), EX_h(e), EX_h2o(e), EX_succ(e), ACALD, ACALDtex, ACALDtpp, DHAPT, ENO, F6PA, FRD2, FUM, GAPD, GLCabcpp, GLCtexi, H2Otex, H2Otpp, HEX1, Htex, MDH, NADH17pp, PDH, PGI, PGK, PGM, PPC, SUCCt3pp, SUCCtex, TPI

9, EX_acald(e), EX_glc(e), EX_h(e), EX_h2o(e), EX_succ(e), ACALD, ACALDtex, ACALDtpp, DHAPT, ENO, F6PA, FRD2, FUM, GAPD, GLCabcpp, GLCtex, H2Otex, H2Otpp, HEX1, Htex, MDH, NADH17pp, PDH, PGI, PGK, PGM, PPC, SUCCt3pp, SUCCtex, TPI

10, EX_acald(e), EX_glc(e), EX_h(e), EX_h2o(e), EX_succ(e), ACALD, ACALDtex, ACALDtpp, ENO, FBA, FRD3, FUM, GAPD, GLCptspp, GLCtexi, H2Otex, H2Otpp, Htex, MDH, NADH18pp, PDH, PFK, PGI, PGK, PGM, PPC, PPCK, SUCCt3pp, SUCCtex, TPI

11, EX_acald(e), EX_glc(e), EX_h(e), EX_h2o(e), EX_succ(e), ACALD, ACALDtex, ACALDtpp, DHAPT, ENO, F6PA, FRD2, FUM, GAPD, GLCabcpp, GLCtex, H2Otex, H2Otpp, HEX7, Htex, MDH, NADH17pp, PDH, PGK, PGM, PPC, SUCCt3pp, SUCCtex, TPI, XYLI2

12, EX_acald(e), EX_glc(e), EX_h(e), EX_h2o(e), EX_succ(e), ACALD, ACALDtex, ACALDtpp, ATPM, ENO, FBA, FRD3, FUM, GAPD, GLCptspp, GLCtexi, H2Otex, H2Otpp, Htex, MDH, NADH18pp, PDH, PFK, PGI, PGK, PGM, PPC, SUCCt3pp, SUCCtex, TPI

13, EX_acald(e), EX_glc(e), EX_h(e), EX_h2o(e), EX_succ(e), ACALD, ACALDtex, ACALDtpp, DHAPT, ENO, F6PA, FRD3, FUM, GAPD, GLCabcpp, GLCtexi, H2Otex, H2Otpp, HEX7, Htex, MDH, NADH18pp, PDH, PGK, PGM, PPC, SUCCt3pp, SUCCtex, TPI, XYLI2

14, EX_acald(e), EX_glc(e), EX_h(e), EX_h2o(e), EX_succ(e), ACALD, ACALDtex, ACALDtpp, ATPM, ENO, FBA, FRD3, FUM, GAPD, GLCptspp, GLCtex, H2Otex, H2Otpp, Htex, MDH, NADH18pp, PDH, PFK, PGI, PGK, PGM, PPC, SUCCt3pp, SUCCtex, TPI

15, EX_acald(e), EX_glc(e), EX_h(e), EX_h2o(e), EX_succ(e), ACALD, ACALDtex, ACALDtpp, ATPM, ENO, FBA, FRD2, FUM, GAPD, GLCptspp, GLCtex, H2Otex, H2Otpp, Htex, MDH, NADH17pp, PDH, PFK, PGI, PGK, PGM, PPC, SUCCt3pp, SUCCtex, TPI

16, EX_acald(e), EX_glc(e), EX_h(e), EX_h2o(e), EX_succ(e), ACALD, ACALDtex, ACALDtpp, ENO, FBA, FBP, FRD2, FUM, GAPD, GLCptspp, GLCtex, H2Otex, H2Otpp, Htex, MDH, NADH17pp, PDH, PFK, PGI, PGK, PGM, PPC, SUCCt3pp, SUCCtex, TPI

17, EX_acald(e), EX_glc(e), EX_h(e), EX_h2o(e), EX_succ(e), ACALD, ACALDtex, ACALDtpp, ENO, FBA, FRD2, FUM, GAPD, GLCptspp, GLCtexi, H2Otex, H2Otpp, Htex, MDH, NADH17pp, PDH, PFK, PGI, PGK, PGM, PPC, PPCK, SUCCt3pp, SUCCtex, TPI

18, EX_acald(e), EX_glc(e), EX_h(e), EX_h2o(e), EX_succ(e), ACALD, ACALDtex, ACALDtpp, ENO, FBA, FBP, FRD3, FUM, GAPD, GLCptspp, GLCtex, H2Otex, H2Otpp, Htex, MDH, NADH18pp, PDH, PFK, PGI, PGK, PGM, PPC, SUCCt3pp, SUCCtex, TPI

19, EX_acald(e), EX_glc(e), EX_h(e), EX_h2o(e), EX_succ(e), ACALD, ACALDtex, ACALDtpp, ENO, FBA, FRD3, FUM, GAPD, GLCptspp, GLCtex, H2Otex, H2Otpp, Htex, MDH, NADH18pp, PDH, PFK, PGI, PGK, PGM, PPC, PPCK, SUCCt3pp, SUCCtex, TPI

20, EX_acald(e), EX_glc(e), EX_h(e), EX_h2o(e), EX_succ(e), ACALD, ACALDtex, ACALDtpp, ENO, FBA, FRD2, FUM, GAPD, GLCptspp, GLCtexi, H2Otex, H2Otpp, Htex, MDH, NADH17pp, NTP1, PDH, PFK, PGI, PGK, PGM, PPC, SUCCt3pp, SUCCtex, TPI

21, EX_acald(e), EX_glc(e), EX_h(e), EX_h2o(e), EX_succ(e), ACALD, ACALDtex, ACALDtpp, ENO, FBA, FBP, FRD2, FUM, GAPD, GLCptspp, GLCtexi, H2Otex, H2Otpp, Htex, MDH, NADH17pp, PDH, PFK, PGI, PGK, PGM, PPC, SUCCt3pp, SUCCtex, TPI

22, EX_acald(e), EX_glc(e), EX_h(e), EX_h2o(e), EX_succ(e), ACALD, ACALDtex, ACALDtpp, ENO, FBA, FRD2, FUM, GAPD, GLCptspp, GLCtex, H2Otex, H2Otpp, Htex, MDH, NADH17pp, NTP1, PDH, PFK, PGI, PGK, PGM, PPC, SUCCt3pp, SUCCtex, TPI

23, EX_acald(e), EX_glc(e), EX_h(e), EX_h2o(e), EX_succ(e), ACALD, ACALDtex, ACALDtpp, ENO, FBA, FRD3, FUM, GAPD, GLCptspp, GLCtexi, H2Otex, H2Otpp, Htex,

MDH, NADH18pp, NTP1, PDH, PFK, PGI, PGK, PGM, PPC, SUCCt3pp, SUCCtex, TPI

24, EX_acald(e), EX_glc(e), EX_h(e), EX_h2o(e), EX_succ(e), ACALD, ACALDtex, ACALDtpp, ENO, FBA, FRD3, FUM, GAPD, GLCptspp, GLCtex, H2Otex, H2Otpp, Htex, MDH, NADH18pp, NTP1, PDH, PFK, PGI, PGK, PGM, PPC, SUCCt3pp, SUCCtex, TPI
